# Ebola virus disease-related stigma among survivors declined in Liberia over an 18-month, post-outbreak period: An observational cohort study

**DOI:** 10.1371/journal.pntd.0007185

**Published:** 2019-02-27

**Authors:** J. Daniel Kelly, Sheri D. Weiser, Barthalomew Wilson, Joseph B. Cooper, Meekie Glayweon, Michael C. Sneller, Clara Drew, Wayne T. Steward, Cavan Reilly, Kumblytee Johnson, Mosoka P. Fallah

**Affiliations:** 1 Department of Medicine, University of California, San Francisco, San Francisco, CA, United States of America; 2 U.S. Partnership for Research on Ebola Virus in Liberia (PREVAIL), Monrovia, Liberia; 3 Laboratory of Immunoregulation, National Institute of Allergy and Infectious Diseases, Bethesda, MD, United States of America; 4 Division of Biostatistics, University of Minnesota, Minneapolis, MN, United States of America; 5 National Institute of Public Health, Ministry of Health, Monrovia, Liberia; University of Texas Medical Branch / Galveston National Laboratory, UNITED STATES

## Abstract

**Background:**

While qualitative assessments of Ebola virus disease (EVD)-related stigma have been undertaken among survivors and the general public, quantitative tools and assessment targeting survivors have been lacking.

**Methods and findings:**

Beginning in June 2015, EVD survivors from seven Liberian counties, where most of the country’s EVD cases occurred, were eligible to enroll in a longitudinal cohort. Seven stigma questions were adapted from the People Living with HIV Stigma Index and asked to EVD survivors over the age of 12 at initial visit (median 358 days post-EVD) and 18 months later. Primary outcome was a 7-item EVD-related stigma index. Explanatory variables included age, gender, educational level, pregnancy status, post-EVD hospitalization, referred to medical care and EVD source. Proportional odds logistic regression models and generalized linear mixed-effects models were used to assess stigma at initial visit and over time. The stigma questions were administered to 859 EVD survivors at initial visit and 741 (86%) survivors at follow-up. While 63% of survivors reported any stigma at initial visit, only 5% reported any stigma at follow-up. Over the 18-month period, there was a significant decrease in stigma among EVD survivors (Adjusted Odds Ratio [AOR], 0.02; 95% Confidence Interval [CI], 0.01–0.04). At initial visit, having primary, junior high or vocational education, and being referred to medical care was associated with higher odds of stigma (educational level: AOR, 1.82; 95%CI, 1.27–2.62; referred: AOR, 1.50; 95%CI, 1.16–1.94). Compared to ages of 20–29, those who had ages of 12–19 or 50+ experienced lower odds of stigma (12–19: AOR, 0.32; 95%CI, 0.21–0.48; 50+: AOR, 0.58 95%CI, 0.37–0.91).

**Conclusions:**

Our data suggest that EVD-related stigma was much lower more than a year after active Ebola transmission ended in Liberia. Among survivors who screened negative for stigma, additional probing may be considered based on age, education, and referral to care.

## Author summary

Survivors of Ebola virus disease (EVD) experienced stigma throughout the 2013–2016 West African outbreak, but post-outbreak experiences of EVD-related stigma have been limited to qualitative studies. We adapted a 7-item EVD-related stigma index from the HIV literature, which was administered to EVD survivors of the observational cohort, Ebola Natural History Study, in Liberia beginning in June 2015. While 63% of 859 EVD survivors reported any EVD-related stigma during the end of the outbreak in Liberia, only 5% of 741 survivors reported any stigma 18-months post-outbreak, suggesting that survivors experienced little to no EVD-related stigma in the absence of active Ebola virus transmission. Among survivors who screened negative for stigma, additional probing may be considered based on age, education, and referral to care.

## Introduction

Ebola virus disease (EVD)-related stigma was a recognized but poorly understood consequence of Ebola outbreaks prior to the 2013 index case of EVD in Guinea.[1–3] The 2013–2016 West Africa EVD outbreak was unprecedented in magnitude; of the 28,646 reported cases, 17,323 individuals survived EVD.[4] This major Ebola outbreak in West Africa was situated in a post-conflict setting marked by a weak health system and mistrust in political actions.[5, 6] The general population lacked education about EVD, and certain messages (e.g., no cure from EVD) heightened fear of EVD and contributed to EVD-related stigma among communities.[7–9] Reintegration of EVD survivors into communities was challenging, particularly early in the outbreak.[10, 11] As a result, Ebola suspects who would have otherwise been diagnosed with EVD evaded quarantines and health systems due to distrust and fear of stigmatization, potentially propagating Ebola virus transmission.[6, 12–15]

During August 2014 in Sierra Leone, a nationally representative study found that 96% of community members had at least one discriminatory attitude toward EVD survivors.[8] EVD survivors suffered from stigma-related issues such as social isolation, gender-based violence,[16] loss of jobs,[17] psychological distress and other problems,[10, 17, 18] in addition to the compounding issues of post-EVD clinical sequelae such as uveitis, muscle pain and memory loss.[19–21] As the outbreak slowed in December 2014 and July 2015, 38–41% of community members still reported at least one discriminatory attitude towards survivors.[8] Of the articles published about EVD survivors from January to March 2016 in the Liberian Daily Observer, 43% explicitly mentioned the word ‘stigma,’ which suggests the persistence of EVD-related stigma at the end of the outbreak in Liberia.[22] The experience of EVD-related stigma among survivors during the post-outbreak period is less known.

A few qualitative studies have described how survivors perceive EVD-related stigma.[23] This research supports Goffman’s definition of stigma as an ‘attribute that is deeply discrediting’ and one that prevents social reintegration.[24] When these negative attitudes are projected upon individuals, they may feel tainted and discounted.[25] As a result, these individuals may anticipate discrimination or other negative acts because they perceive a social identity that is devalued.[26, 27] Indeed, there has been a history of stigma occurring in settings of emerging infectious and deadly diseases, not just for EVD.[28, 29] Translating the conceptual framework of stigma into a disease-specific stigma scale, however, is a lengthy process that has generally not been possible in emergency settings, as was the case during the West African EVD outbreak. To assess EVD-related stigma and discriminatory attitudes towards West African survivors, one research group (Focus 1000) adapted HIV stigma questions from the 2013 Demographic and Health Survey in Sierra Leone that were administered to community members.[8, 9, 30] However, there were no scales developed or adapted to assess how survivors perceive and experience EVD-related stigma and discrimination. As a result, quantitative studies of EVD-related stigma among survivors are lacking.

Starting in June of 2015, the Partnership for Research on Ebola Virus in Liberia (PREVAIL) initiated a 5-year Ebola Natural History Study of EVD survivors (PREVAIL-III). One aspect of this study assessed EVD-related stigma among survivors using an adapted quantitative data collection tool from the People Living with HIV (PLHIV) Stigma Index.[31] As a result, this study offers a unique opportunity to determine the predictors and trend of EVD-related stigma over time in the largest cohort of EVD survivors under investigation in West Africa. Despite persistent reports of EVD-related stigma and discriminatory attitudes among community members towards survivors, we hypothesized that EVD-related stigma among survivors will resolve in the years after the outbreak, except for pregnant women, women of reproductive age, and HIV-positive individuals. These sub-groups, particularly pregnant women, were thought to have asymptomatic viral shedding that could co-occur during exposure to vaginal or other bodily fluids while these individuals were in an immunocompromised state.[32] Women of reproductive age were anecdotally observed to be affected by EVD-related stigma, by proxy of pregnant women and lactating mothers.[33] Understanding the changes and predictors of stigma during the post-outbreak period has the potential to identify high-risk individuals and inform organizations of potential interventions over time.

## Methods

### Ethics statement

The National Research Ethics Board of Liberia and the National Institute of Allergy and Infectious Diseases Institutional Review Board (IRB) at the United States National Institutes of Health approved the study protocol. Before any study-related procedures were conducted, participants signed or marked the approved informed consent form, and parents or guardians provided this consent on behalf of all child participants. A video and picture booklets that describe the study were used to ensure that illiterate volunteers understand the study requirements and risks and benefits.

### Study design and participants

Beginning in June 2015, EVD survivors were eligible to enroll in the Ebola Natural History Study (PREVAIL-III). PREVAIL-III used a social mobilization team to create awareness on the recruitment processes for the study through stakeholder meetings, community sensitization, media engagements, focus group discussion with Ebola Survivor Network of Liberia and other social groupings through Montserrado, Margibi, Bomi, Cape Mount, Lofa, Nimba, and Grand Bassa Counties. Monrovia, the capital city of Liberia, is situated inside Montserrado County. EVD survivors of any age were included in the study if they were listed in the national registry provided by the Ministry of Health (MOH) and lived in Montserrado, Margibi, Bomi, Cape Mount, Lofa, Nimba, and Grand Bassa Counties of Liberia. These seven Counties were where most of the Liberia’s EVD cases occurred.[4]

At enrollment, PREVAIL-III study staff administered a questionnaire, conducted a physical examination, and obtained blood samples from each participant. Participants subsequently had a study visit approximately every 6 months. At the end of every study visit, the study physician decided if the current health status of the participant required clinical care. PREVAIL did not provide clinical care, except for ocular inflammatory disease, so the study physician referred these participants to a partnered healthcare facility for further evaluation.

It is important to note that prior to the start of PREVAIL-III, the EVD-related stigma measurement underwent a development process that occurred during the first half of 2015. It was then launched later that year (June 2015). The initial visit captured experiences of stigma over a period of active Ebola virus transmission. In addition to the initial visit, the EVD-related stigma index was included in the questionnaire at the fourth study visit, 18 months later. Interviewers administered the questions to participating EVD survivors 12 years and older. Note that after the initial visit, the stigma questions were removed from subsequent study visits until it was hypothesized that EVD-related stigma may be declining. In order to evaluate the extent to which stigma may have been changing over time, the stigma questions were repeated at the fourth study visit.

### Measurement

Our primary outcome was EVD-related stigma, measured by a 7-item index adapted from the PLHIV Stigma Index.[31] Since 2008, the PLHIV Stigma Index has been administered to over 100,000 PLHIV in more than 90 countries, including Liberia, and has been translated into 54 languages. The PLHIV Stigma Index was administered in Liberia in 2013 and included 63 items and 9 parts (S1 Appendix). The following parts of the index included items that were plausibly related to EVD-related stigma: 13 items about stigma and discrimination from other people, 9 items about access to work and health and education services; and 4 items about internalized stigma and fears. The following parts of the PLHIV Stigma Index were broadly evaluated not to be applicable to EVD-related stigma: 6 items about rights, laws, and policies; 10 items about effecting change; 3 items about testing and diagnosis; 6 items about disclosure and confidentiality; 5 items about treatment; and 7 items about having children.

Given that there was broad agreement that EVD survivors were facing stigma and discrimination, and the PLHIV Stigma Index had already been adapted and used in Liberia, this tool was selected as the parent index from which questions would be adapted for assessing EVD-related stigma. The entire PLHIV Stigma Index was reviewed and discussed with a group of EVD survivors who were enrolled in the PREVAIL-III study. Study staff took notes on the discussion with the goal of identifying a group of items from the PLHIV Stigma Index that could be adapted to EVD-related stigma items. This discussion occurred during a routine psychosocial counseling meeting; over fifty EVD survivors attended the meeting. Although only a majority was requested to reach consensus, nearly all attendees of the EVD survivor meeting agreed upon seven items and the adaptations.

The seven stigma items, listed in Table A in S2 Appendix, were considered relevant to the experience of EVD survivors. Two items were selected from the sub-index about stigma and discrimination from other people, two items were selected from the sub-index about access to work and health and education services, and three items were selected from the sub-index about internalized stigma. Instead of using Likert responses, participants advised that the EVD-related stigma index offer binary responses (yes/no). In addition, the language was culturally adapted to the experiences of EVD survivors. The items were framed to capture any occurrences of stigma over the time period prior to the previous study visit. At the initial visit, participants were asked to recall experiences of stigma since discharge (approximately twelve months), and at visit 4, participants were asked to recall experiences of stigma in the prior six months.

These adaptations formed the basis for the EVD-related stigma index, which was considered an ordinal variable with values ranging from 0 to 7. Dr. Mosoka Fallah, the Liberian PREVAIL-III Principal Investigator, and members of the Carter Center, which managed a mental health program for EVD survivors in Liberia, reviewed the items and adaptations prior to use.

The EVD-related stigma index was defined as the sum of affirmative responses with a maximum score of seven and a minimum score of zero. The estimated Cronbach’s alpha for the EVD-related stigma index was 0.60 at initial visit (95% Confidence Interval (CI), 0.55–0.65) and 0.57 at follow-up (95% CI, 0.50–0.63), indicating an acceptable degree of internal reliability. We also conducted a confirmatory factor analysis and identified two potential sub-indices. These sub-indices, however, did not have an acceptable degree of internal reliability and were excluded from statistical analyses.

Independent variables obtained through the questionnaire at the initial visit were selected based on potential associations between EVD-related stigma and EVD survivors who have significant interactions with the community and health system due to their age, job, education, or experience with the health system. Variables included age, gender, educational level, pregnancy status, hospitalization since acute EVD illness, and source of Ebola virus infection. These variables represented events that occurred prior to initial visit of EVD-related stigma. Each study visit, the variable ‘being referred to medical care’ described whether participants who were evaluated by study physicians were given a referral form for medical care for any non-ocular health issue(s) (yes/no). While the participant was ‘referred to medical care’ during the study visit, the health issue prompting the referral was considered to be an event that occurred prior to the evaluation for EVD-related stigma.

## Statistical analyses

Descriptive characteristics of participants were calculated at the initial visit and the fourth study visit and compared to assess for group differences between visits. Age (12–19; 20–29; 30–39; 40–49; 50+), education (no formal education; primary, junior high or vocational; high school or beyond), and source of Ebola virus infection (family; job related; other/unknown) were categorized, and the other variables were dichotomized. We used generalized linear mixed-effects models and stigma measures from both visits to analyze the relationship between the odds of experiencing any stigma (yes/no) and being a member of certain subgroups. Subgroups included pregnant women, women of reproductive age, and those with HIV-positive serostatus. In these models, a random effect for participant ID was used to account for the correlation within subject. To examine effects of certain predictors on stigma levels at the initial visit, we fit a proportional odds logistic regression model. Age, gender, education, hospitalization, being referred to medical care, pregnancy, and Ebola virus infectious source were used as predictors of the odds of higher EVD-related stigma. We conducted sensitivity analyses that tested different types of regression models, including linear (Table B in S2 Appendix) and Poisson models (Table C in S2 Appendix), and assessed individual stigma items for potentially biased associations with predictors (Tables D-F in S2 Appendix). We also ran a regression excluding HIV positive participants (Table G in S2 Appendix). Finally, we also investigated potential collinearity between our predictors. Variance inflation factors are reported in Tables H and I in S2 Appendix. These sensitivity analyses showed results consistent with those reported in this paper and supported use of our final model. Analyses were performed using STATA/IC 13.1 (STATA Corporation, College Station, TX) and R (Version 3.2.3) including packages MASS, dplyr, car, psych and xtable.

## Results

At the initial visit, 1,074 EVD survivors were enrolled, and the EVD-related stigma questionnaire was administered to all EVD survivors aged 12 or older. We excluded participants who were enrolled as survivors and had antibodies inconsistent with prior Ebola virus infection and those under 12 years who were wrongfully administered the survey. This left a total of 859 participants. The initial visit occurred median 358 days from discharge from an Ebola Treatment Unit (interquartile range: 310, 403). A follow-up measurement of EVD-related stigma was obtained from 741 (86%) of 859 EVD survivors during visit 4 of the PREVAIL III study. Characteristics of the EVD survivors at the initial visit and visit 4 are summarized in **Table 1.** With the exception of ‘being referred to medical care,’ there were no differences between groups at initial visit and at visit 4.

**Table 1:** Summary of demographics for survivors at initial visit and visit 4.

	Initial Assessment	Visit 4 (18 months)	p-value
	N = 859	N = 741	
Female	56%	57.1%	0.7
Age 12–19	16.4%	16.3%	
Age 20–29	28.5%	29%	
Age 30–39	26.9%	27.1%	1.00
Age 40–49	16.8%	16.3%	
Age 50+	11.4%	11.2%	
Education (No formal education)^1^	20.6%	19.3%	
Education (Primary, junior high or vocational)^1^	38%	38.2%	0.81
Education (High school or beyond)^1^	41.4%	42.4%	
Pregnant^2^	13.6%	8.6%	0.03
Woman of Reproductive Age	45.4%	47.1%	0.53
HIV Positive	1.4%	2%	0.45
Hospitalized Since Acute EVD^3^	3.3%	3.2%	1.00
Referred to Medical Care	41.2%	12.1%	<0.005
Infection Source (Family)^4^	75.8%	76.5%	
Infection Source (Job related)^4^	7.9%	7.7%	0.93
Infection Source (Other/unknown)^4^	16.3%	15.7%	

1. Education is only measured at initial assessment so changes in percentages show distribution of loss to follow-up.

2. Pregnancy percentage is measured out of women of reproductive age.

3. Hospitalization since Acute EVD is only measured at initial assessment so changes in percentage show distribution of loss to follow-up.

4. Infection source is only measured at baseline so changes in percentages show distribution of loss to follow-up.

### Description of EVD-related stigma

At the initial visit, the majority (63%) of EVD survivors reported at least one item from the EVD-related stigma index. The most commonly reported experiences of stigma were job or income loss (35.2%) and forced relocation due to social alienation (24.8%). By contrast, there were relatively fewer individuals reporting that they withdrew from education/training or did not take up an opportunity (12.1%), were deprived from attending a gathering (11.3%), or lost a spouse due to fears of infection (8.4%). At visit 4, few (5%) EVD survivors reported at least one item from the EVD-related stigma index. These experiences included forced relocation (3.5%), isolating behaviors (2.0%), and job or income loss (1.3%). Other experiences were extremely rare or not reported at visit 4 (**Table 2**).

**Table 2:** Summary of stigma endorsed by survivors at initial visit and visit 4.

	Initial Assessment	Visit 4 (18 months)
	N = 859	N = 741
Forced to change residence because of social alienation from family?	213 (24.8%)	26 (3.5%)
Lost a job or another source of income because of being infected?	302 (35.2%)	10 (1.3%)
Lost a spouse because of fear of being infected?	72 (8.4%)	5 (0.7%)
Deprived from attending gathering (e.g., school, church, social)?	97 (11.3%)	3 (0.4%)
Isolated yourself from family and/or friends?	174 (20.3%)	15 (2%)
Withdrew from education/training or did not take up an opportunity?	104 (12.1%)	0 (0%)
Afraid that someone would not want to be sexually intimate?	188 (21.9%)	3 (0.4%)

### Changes in EVD-related stigma over time

Between initial visit and visit 4, there was a significant decrease in EVD-related stigma among the EVD survivors (Adjusted Odds Ratio [AOR], 0.02; 95% Confidence Interval [CI], 0.01–0.04; p<0.005). There was no difference in changes of EVD-related stigma among pregnant women, women of reproductive age and HIV-positive persons over the 18-month period (**Table 3**).

**Table 3:** Longitudinal associations between stigma and all survivors as well as survivors who were pregnant, women of reproductive age, or HIV-positive.

	Estimated Odds Ratio (95% CI)	p-value
18-Month Visit	0.02 (0.01, 0.04)	<0.005
Pregnant	0.79 (0.43, 1.48)	0.47
Woman of Reproductive Age	1.3 (0.96, 1.75)	0.09
HIV Positive	0.83 (0.26, 2.71)	0.76

### Associations with EVD-related stigma at initial visit

Compared to not having any formal education, having a primary, junior high or vocational school education was associated with higher odds of stigma (AOR, 1.82; 95% CI, 1.27–2.62; p<0.005). Additional education was not significantly associated with higher stigma odds. Compared to ages of 20–29, those who had ages of 12–19 or 50+ experienced lower odds of stigma (12–19: AOR, 0.32; 95%CI, 0.21–0.48; 50+: AOR, 0.58 95%CI, 0.37–0.91). Survivors who were referred to medical care during their initial visit had higher odds of stigma than those who were not referred (AOR, 1.50; 95% CI, 1.16–1.94; p<0.005). Gender, hospitalized since acute EVD, pregnancy, and Ebola virus source were not associated with odds of stigma (**Table 4**).

**Table 4:** Associations between predictors and stigma at initial visit.

	Estimated Odds Ratio (95% CI)	p-value
Education (Primary, junior high or vocational)^1^	1.82 (1.27, 2.62)	<0.005
Education (High school or beyond)^1^	1.2 (0.84, 1.73)	0.32
Age (12–19)^2^	0.32 (0.21, 0.48)	<0.005
Age (30–39)^2^	1.15 (0.83, 1.61)	0.39
Age (40–49)^2^	0.91 (0.61, 1.33)	0.61
Age (50+)^2^	0.58 (0.37, 0.91)	0.02
Gender (Female)	1.16 (0.89, 1.52)	0.28
Hospitalized Since Acute EVD	1.19 (0.59, 2.35)	0.63
Referred to Medical Care	1.5 (1.16, 1.94)	<0.005
Pregnant	0.79 (0.47, 1.33)	0.38
Infection Source (Family)^3^	1.06 (0.76, 1.5)	0.72
Infection Source (Job related)^3^	1.42 (0.83, 2.42)	0.2

1. No formal education is comparison group for education.

2. Age 20–29 is comparison group for age.

3. Other/unknown is the comparison group for infection source.

## Discussion

To the best of our knowledge, this is the first quantitative assessment of EVD-related stigma among survivors. Communities held high levels of discriminatory attitudes towards EVD survivors early in the outbreak and moderately elevated levels of discriminatory attitudes persisted through the end of the outbreak.[8] After the outbreak, reports of EVD-related stigma among EVD survivors declined. Furthermore, there was not strong evidence that persistent stigma occurred among marginalized populations such as pregnant women, women of reproductive age, and HIV-infected persons. We encourage survivor support programs to identify and attend to the small minority of EVD survivors who faced persistent stigma, even in the absence of active Ebola virus transmission. Although EVD-related stigma may not be an ongoing issue of public health significance in West Africa, it may be relevant to the current EVD outbreak in North Kivu, Democratic Republic of Congo.[34] Outbreak programs should continue to consider stigma in their preparedness and response strategies since stigma is a social construct that may re-emerge during any EVD outbreak.

Survivors who had an age of 20–49, had some formal education, and were referred to medical care had an increased risk for stigma. In-depth assessments with probing may be considered in these higher risk populations, particularly among EVD survivors who screen negative for stigma. Screening all EVD survivors for stigma is important, though, because any survivor may experience stigma and all of the West African survivors were eligible for anti-stigma support services, including psychosocial counseling. Although we found that stigma declined among most survivors, these survivors may continue to suffer from other mental illness (e.g., anxiety) and have a need for ongoing support services. [15] A baseline report from a national program assessment found that almost all EVD survivors utilized mental health services.[17]. Moreover, the social mobilization team for the PREVAIL-III study conducted community-level and individual-level anti-stigma interventions for the first year of the study period. It is possible that multiple factors contributed to the decline of stigma among EVD survivors, including the end of the epidemic, resolving post-EVD clinical sequelae, and anti-stigma interventions. However, data on anti-stigma interventions in Ebola survivors using an EVD-related stigma assessment tool are lacking from the literature. In future Ebola outbreaks, formal program evaluations of anti-stigma interventions are needed.

This study adapted stigma questions from the PLHIV Stigma Index to measure EVD-related stigma.[31] Although these questions were selected during the emergency response to the Ebola outbreak, the 7-item stigma index demonstrated internal reliability and good performance in this study. During the outbreak, EVD survivors experienced stigma as a consequence of community-level discriminatory attitudes,[8, 9] so it is possible that if another EVD outbreak were to occur, stigma may recur. While our EVD-related stigma index may require additional validation studies, we encourage its use during future EVD outbreaks, particularly to identify stigmatized survivors and evaluate anti-stigma interventions. We may also consider adapting use of this EVD-specific stigma index to other non-EVD outbreak settings, given that there are qualitative reports of Marburg virus survivors suffering from stigma.[29]

This study has limitations. The EVD-related stigma index was not formally developed and may not be a completely comprehensive measure of stigma. Because PREVAIL-III was established in the setting of an emergency and the HIV stigma literature offered potentially adaptable measurement tools, the construct validities of the index were considered acceptable for the circumstances. Findings are specific to the time period between latter half of the Ebola outbreak and 18 months after the outbreak. Participants were asked to recall a longer period at the initial visit (12 months) than at visit 4 (6 months). The shorter recall period at visit 4 may have contributed to a lower amount of self-reported stigma. However, the difference between self-reported stigma at these visits (63% at initial visit; 5% at visit 4) was large enough that the decline in stigma was unlikely to be a Type-1 error. Given the low level of stigma at visit 4, we were unable to use repeated measures of stigma to identify temporally associated risk factors. As a result, the explanatory variables and stigma outcomes were assessed at the initial visit, so reversal causality could be another threat to validity. The setting of the study population was the greater Monrovia area, which is urban and peri-urban, meaning that these findings are not generalizable to rural or other settings. The study was not powered to assess sub-groups of pregnant women, women of reproductive age or HIV-positive individuals. Findings related to EVD-related stigma may be different in Sierra Leone, Guinea, and other settings where there were Ebola outbreaks. Despite the limitations, the large sample size, longitudinal design, and significant findings provide an important lens into EVD-related stigma.

Within 18 months after the end of the Ebola outbreak in Liberia, most EVD survivors reported little to no EVD-related stigma. Before the 2013–2016 West Africa outbreak,[1, 2] EVD-related stigma existed and the West Africa outbreak offered an opportunity to advance our understanding of this stigma.[22, 23] While age, education, and referral to medical care may be used to screen for EVD survivors who may benefit from early anti-stigma interventions, qualitative study may improve our understanding of persistent stigma. In future Ebola outbreaks, the novel measurement tool described in this study will be available to assess EVD-related stigma and may be part of strategies to intervene upon this modifiable social process.

## Supporting information

S1 Checklist STROBE checklist. (DOCX)

S1 Appendix Survey instrument for people living with HIV (PLHIV) stigma. (DOCX)

S2 Appendix Survey instrument for EVD-related stigma, sensitivity analyses, and multicollinearity assessment. (DOCX)
