# Supplementary material for: Ebola virus disease-related stigma among survivors declined in Liberia over an 18-month, post-outbreak period: An observational cohort study
Source: PLoS Negl Trop Dis. 2019 Feb 27;13(2):e0007185. doi: 10.1371/journal.pntd.0007185 (PMC6411197; doi:10.1371/journal.pntd.0007185)
Supplement: S1 Appendix — (DOCX) [file pntd.0007185.s002.docx]

S1 Appendix. Survey instrument for People Living with HIV (PLHIV) stigma.

PEOPLE LIVING WITH HIV STIGMA INDEX: THE QUESTIONNAIRE

#### CONFIDENTIAL AND ANONYMOUS

**Before *starting* the interview, you must:**

1. **Give the interviewee the information sheet and allow him/her time to read through it. If he/she is unable to read, you must read it out to him/her.**
2. **Read the informed consent form to the interviewee. If he/she agrees to participate in the study, complete two copies of the form. After both forms have been signed, give one to the interviewee for him/her to keep and you keep the other one.**

**On *finishing* the interview, please complete the following:**

**REFERRALS AND FOLLOW-UP**

| 1. | Did the interviewee need a referral? | | | Yes | ❒_1_ | No | | ❒_2_ |
| --- | --- | --- | --- | --- | --- | --- | --- | --- |
| 2. | If **Yes**, what kind of referral(s)? | | Legal | | | | ❒_1_ | |
|  |  | Counselling | | | | | ❒_2_ | |
|  |  | Support group | | | | | ❒_3_ | |
|  |  | Other | | | | | ❒_4_ | |
|  | If **Other**, where did you refer them to? | | | | | |  | |
| 3. | What steps have you taken to help the interviewee with the above referral(s)?  (Tick more than one box if appropriate). | | | | | |  | |
|  |  | I have given sufficient information on the referral(s) already | | | | | ❒_1_ | |
|  |  | I will send the required information to the interviewee | | | | | ❒_2_ | |
|  |  | Further follow-up is needed | | | | | ❒_3_ | |

Please give details of what you promised to do about referral(s) after the interview, if anything:

|  |
| --- |

| 4. | Is this interviewee a potential candidate for a case study? | | Yes | ❒_1_ | No | | ❒_2_ |
| --- | --- | --- | --- | --- | --- | --- | --- |
| 5. | If **Yes**, record the time and date of the case study meeting: | Time: |  | Date: | |  | |

**QUALITY CONTROL PROCEDURES:**

**Control panel – to be filled in *only* when your task* has been completed.**

|  | **Name** | **Signature** | **Date** |
| --- | --- | --- | --- |
| Interviewer |  |  |  |
| Team leader |  |  |  |
| Data entry 1 |  |  |  |
| Data entry 2 |  |  |  |

***Tasks:**

- The **interviewer** must ensure that all sections of the questionnaire are completed properly and in full, unless the interviewee does not wish to complete them – in which case this must be noted alongside the relevant question(s).
- The **team leader** must check the questionnaire carefully and query any apparent discrepancies with the interviewer. The **quality checks** section at the end of this questionnaire will help the interviewer and team leader with these tasks.
- **Data entry people 1 and 2** must enter all data from the questionnaire correctly. They must enter the data from every questionnaire independently, following the procedures outlined in the **user guide.**

***Section 1: Information about you***

| 1. Sex | Male ❒_1_ | Female ❒_2_ | Transgender ❒_3_ |
| --- | --- | --- | --- |

| 2. How old are you? |  |  |
| --- | --- | --- |
|  | Youth aged 15 to 19 years | ❒_1_ |
|  | Adult aged 20 – 24 years | ❒_2_ |
|  | Adult aged 25 – 29 years | ❒_3_ |
|  | Adult aged 30 - 39 years | ❒_4_ |
|  | Adult aged 40 – 49 years | ❒_5_ |
|  | Adult aged 50 + years | ❒_6_ |

| 3. For how long have you been living with HIV? (Tick one box only). |  |  |
| --- | --- | --- |
|  | 0 - 1 year | ❒_1_ |
|  | 1 - 4 years | ❒_2_ |
|  | 5 - 9 years | ❒_3_ |
|  | 10 - 14 years | ❒_4_ |
|  | 15 + years | ❒_5_ |

| 4. Current relationship status (Tick one box only). |  |
| --- | --- |
| Married or cohabiting and husband/wife/partner is currently living in household | ❒_1_ |
| Married or cohabiting but husband/wife/partner is temporarily living/working away  from the household | ❒_2_ |
| In a relationship but not living together | ❒_3_ |
| Single | ❒_4_ |
| Divorced/separated | ❒_5_ |
| Widow/widower | ❒_6_ |

| 5. If you are currently in a relationship, for how long have you been involved with your husband/wife/partner? |  |  |
| --- | --- | --- |
|  | 0 - 1 year | ❒_1_ |
|  | 1 - 4 years | ❒_2_ |
|  | 5 - 9 years | ❒_3_ |
|  | 10 - 14 years | ❒_4_ |
|  | 15 + years | ❒_5_ |

| 6. Are you sexually active at the moment? | Yes ❒_1_ | No ❒_2_ |
| --- | --- | --- |

| 7. Do you belong to, or have you in the past belonged to, any of the following categories?  (Tick at least one box. You can tick more than one if appropriate). | | | |
| --- | --- | --- | --- |
| Men who have sex with men | ❒_1_ | Internally displaced person | ❒_7_ |
| Gay or lesbian | ❒_2_ | Member of an indigenous group | ❒_8_ |
| Transgender | ❒_3_ | Migrant worker | ❒_9_ |
| Sex worker | ❒_4_ | Prisoner | ❒_10_ |
| Injecting drug user | ❒_5_ | I don’t belong to, and have not in the past belonged to, any of these categories | ❒_11_ |
| Refugee or asylum seeker | ❒_6_ |  |  |

| 8. Do you have a physical disability of any kind (not including general ill health related to HIV)? | Yes ❒_1_ | No ❒_2_ |
| --- | --- | --- |

If **YES**, please describe what this physical disability is below:

|  |
| --- |

| 9. What is the highest level of formal education you have completed?  (Tick one box only). | No formal education ❒_1_  Primary school ❒_2_  Secondary school ❒_3_  Technical college/university ❒_4_ |
| --- | --- |

| 10. Which one of these statements best describes your current employment status?  (Tick at least one box. You can tick more than one if appropriate). | In full-time employment (as an employee) ❒_1_  In part-time employment (as an employee) ❒_2_  Working full-time but not as an employee (self-employed) ❒_3_  Doing casual or part-time work (self-employed) ❒_4_ Unemployed and not working at all ❒_5_ |
| --- | --- |

| 11. How many people currently live in your household in each of these age categories? | |
| --- | --- |
|  | Number of people |
| Children aged 0-14 years |  |
| Youth aged 15-19 years |  |
| Adults aged 20-24 years |  |
| Adults aged 25-29 years |  |
| Adults aged 30-39 years |  |
| Adults aged 40-49 years |  |
| Adults aged 50 years and above |  |

|  | Number of orphans |
| --- | --- |
| 12. How many of the children and youth in your household are AIDS orphans? |  |

| 13. Is your household in:  (Tick one box only). | A rural area ❒_1_  A small town or village ❒_2_  A large town or city ❒_3_ |
| --- | --- |

| 14. What was the average income of your household per month over the last 12 months? (Write down the figure in local currency.) |  |
| --- | --- |
|  |  |
| **For data capturers only:** |  |
| Annual income in local currency: |  |
| Current exchange rate from local currency to US dollars: |  |
| Annual income in US dollars: |  |
|  |  |

| 15. In the last month, how many days has any member of your household not had enough food to eat? |  |
| --- | --- |

***Section 2A: Your experience of stigma and discrimination from other people***

| 1a. In the last 12 months, how often have you been excluded from social gatherings or activities (e.g. weddings, funerals, parties, clubs)?  (Tick one box only). | Never ❒_1_  Once ❒_2_  A few times ❒_3_  Often ❒_4_ |
| --- | --- |

**If the answer is NEVER, please go to question 2a.**

| 1b. If so, was this…? (Tick one box only). | Because of your HIV status?  ❒_1_ | For (an)other reason(s)?  ❒_2_ | Both because of your HIV status and other reason(s)?  ❒_3_ | Not sure why  ❒_4_ |
| --- | --- | --- | --- | --- |

| 2a. In the last 12 months, how often have you been excluded from religious activities or places of worship?  (Tick one box only). | Never ❒_1_  Once ❒_2_  A few times ❒_3_  Often ❒_4_ |
| --- | --- |

**If the answer is NEVER, please go to question 3a.**

| 2b. If so, was this…?(Tick one box only). | Because of your HIV status?  ❒_1_ | For (an)other reason(s)?  ❒_2_ | Both because of your HIV status and other reason(s)?  ❒_3_ | Not sure why  ❒_4_ |
| --- | --- | --- | --- | --- |

| 3a. In the last 12 months, how often have you been excluded from family activities (e.g. cooking, eating together, sleeping in the same room?)  (Tick one box only). | Never ❒_1_  Once ❒_2_  A few times ❒_3_  Often ❒_4_ |
| --- | --- |

**If the answer is NEVER, please go to question 4a.**

| 3b. If so, was this…? (Tick one box only). | Because of your HIV status?  ❒_1_ | For (an)other reason(s)?  ❒_2_ | Both because of your HIV status and other reason(s)?  ❒_3_ | Not sure why  ❒_4_ |
| --- | --- | --- | --- | --- |

| 4a. In the last 12 months, how often have you been aware of being gossiped about?  (Tick one box only). | Never ❒_1_  Once ❒_2_  A few times ❒_3_  Often ❒_4_ |
| --- | --- |

**If the answer is NEVER, please go to question 5a.**

| 4b. If so, was this…? (Tick one box only). | Because of your HIV status?  ❒_1_ | For (an)other reason(s)?  ❒_2_ | Both because of your HIV status and other reason(s)?  ❒_3_ | Not sure why  ❒_4_ |
| --- | --- | --- | --- | --- |

| 5a. In the last 12 months, how often have you been verbally insulted, harassed and/or threatened?  (Tick one box only). | Never ❒_1_  Once ❒_2_  A few times ❒_3_  Often ❒_4_ |
| --- | --- |

**If the answer is NEVER, please go to question 6a.**

| 5b. If so, was this…? (Tick one box only). | Because of your HIV status?  ❒_1_ | For (an)other reason(s)?  ❒_2_ | Both because of your HIV status and other reason(s)?  ❒_3_ | Not sure why  ❒_4_ |
| --- | --- | --- | --- | --- |

| 6a. In the last 12 months, how often have you been physically harassed and/or threatened?  (Tick one box only). | Never ❒_1_  Once ❒_2_  A few times ❒_3_  Often ❒_4_ |
| --- | --- |

**If the answer is NEVER, please go to question 7a.**

| 6b. If so, was this…? (Tick one box only). | Because of your HIV status?  ❒_1_ | For (an)other reason(s)?  ❒_2_ | Both because of your HIV status and other reason(s)?  ❒_3_ | Not sure why  ❒_4_ |
| --- | --- | --- | --- | --- |

| 7a. In the last 12 months, how often have you been physically assaulted?  (Tick one box only). | Never ❒_1_  Once ❒_2_  A few times ❒_3_  Often ❒_4_ |
| --- | --- |

**If the answer is NEVER, please go to question 8.**

| 7b. If so, was this…? (Tick one box only). | Because of your HIV status?  ❒_1_ | For (an)other reason(s)?  ❒_2_ | Both because of your HIV status and other reason(s)?  ❒_3_ | Not sure why  ❒_4_ |
| --- | --- | --- | --- | --- |

| 7c. If so, who physically assaulted you? (Tick one box only.) | My husband/ wife/partner ❒_1_  Another member of the household ❒_2_  Person(s) outside the household who is/are known to me ❒_3_  Unknown person(s) ❒_4_ |
| --- | --- |

| 8. In questions 1 – 7, if you experienced stigma and/or discrimination for reasons **other than your HIV status,** please choose one category that best explains why you felt you were stigmatized and/or discriminated against. (Tick one box only.) | Sexual orientation (men who have sex with men, gay or lesbian, transgender) ❒_1_  Sex worker ❒_2_  Injecting drug user ❒_3_  Refugee or asylum seeker ❒^4^  Internally displaced person ❒^5^  Member of an indigenous group ❒^6^  Migrant worker ❒_7_  Prisoner ❒^8^  None of the above – it was because of an(other) reason(s) ❒^9^ |
| --- | --- |

If you chose **None of the above**, please describe below what you believe the other reason(s) was/were for this in each of these situations.

|  |
| --- |

| 9. In the last 12 months, how often have you been subjected to psychological pressure or manipulation by your husband/wife or partner in which your HIV-positive status was used against you? (Tick one box only). | Never ❒_1_  Once ❒_2_  A few times ❒_3_  Often ❒_4_ |
| --- | --- |

| 10. In the last 12 months, how often have you experienced sexual rejection as a result of your HIV positive status?  (Tick one box only) | Never ❒_1_  Once ❒_2_  A few times ❒_3_  Often ❒_4_ |
| --- | --- |

| 11. In the last 12 months, how often have you been discriminated against by other people living with HIV?  (Tick one box only.) | Never ❒_1_  Once ❒_2_  A few times ❒_3_  Often ❒_4_ |
| --- | --- |

| 12. In the last 12 months, how often has your wife/husband or partner, or any members of your household experienced discrimination as a result of your HIV-positive status?  (Tick one box only.) | Never ❒_1_  Once ❒_2_  A few times ❒_3_  Often ❒_4_ |
| --- | --- |

| 13. If you have experienced some form of HIV-related stigma and/or discrimination in the last 12 months, *why* do you think this is?  (Tick more than one box if appropriate.) | People are afraid of getting infected with HIV from me | ❒_1_ |
| --- | --- | --- |
|  | People don’t understand how HIV is transmitted and are afraid I will infect them with HIV through casual contact | ❒_2_ |
|  | People think that having HIV is shameful and they should not be associated with me | ❒_3_ |
|  | Religious beliefs or “moral” judgements | ❒_4_ |
|  | People disapprove of my lifestyle or behaviour | ❒_5_ |
|  | I look sick with symptoms associated with HIV | ❒_6_ |
|  | I don’t know/I am not sure of the reason(s) | ❒_7_ |

***Section 2B: Your access to work and health and education services***

| 1a. In the last 12 months, how often have you been forced to change your place of residence or been unable to rent accommodation?  (Tick one box only.) | Never ❒_1_  Once ❒_2_  A few times ❒_3_  Often ❒_4_ |
| --- | --- |

| 1b. If so, was this…? (Tick one box only). | Because of your HIV status?  ❒_1_ | For (an)other reason(s)?  ❒_2_ | Both because of your HIV status and other reason(s)?  ❒_3_ | Not sure why  ❒_4_ |
| --- | --- | --- | --- | --- |

**If interviewee has *not* been earning an income (either through some form of formal employment or on a casual or part-time basis) or has not been self-employed during the last 12 months, go to Question 5.**

| 2a. In the last 12 months, how often have you lost a job (if employed) or another source of income (if self-employed or an informal/casual worker)?  (Tick one box only.) | Never ❒_1_  Once ❒_2_  A few times ❒_3_  Often ❒_4_ |
| --- | --- |

| 2b. If so, was this…? (Tick one box only.) | Because of your HIV status?  ❒_1_ | For (an)other reason(s)?  ❒_2_ | Both because of your HIV status and other reason(s)?  ❒_3_ | Not sure why  ❒_4_ |
| --- | --- | --- | --- | --- |

| 2c. If because of HIV status (wholly or partly), did you lose your work/income…?  (Tick one box only). | Because of discrimination by your employer or co-workers ❒_1_  Because you felt obliged to stop working due to poor health ❒_2_  Because of a combination of discrimination and poor health ❒_3_  Because of another reason ❒_4_ |
| --- | --- |

| 3. In the last 12 months, have you been refused employment or a work opportunity because of your HIV status? | Yes ❒_1_ | No ❒_2_ |
| --- | --- | --- |

| 4a. In the last 12 months, how often has your job description or the nature of your work changed, or have you been refused promotion, as a result of your HIV status?  (Tick one box only.) | Never ❒_1_  Once ❒_2_  A few times ❒_3_  Often ❒_4_ |
| --- | --- |

| 4b. If so, did this happen…?  (Tick one box only.) | Because of discrimination by your employer or co-workers ❒_1_  Because poor health prevented you from doing certain things ❒_2_  Because of a combination of discrimination and poor health ❒_3_  Other reason ❒_4_ |
| --- | --- |

| 5. In the last 12 months, how often have you been dismissed, suspended or prevented from attending an educational institution because of your HIV status?  (Tick one box only.) | Never ❒_1_  Once ❒_2_  A few times ❒_3_  Often ❒_4_  Not applicable ❒_5_ |
| --- | --- |

| 6. In the last 12 months, how often has your child/children been dismissed, suspended or prevented from attending an educational institution because of your HIV status?  (Tick one box only.) | Never ❒_1_  Once ❒_2_  A few times ❒_3_  Often ❒_4_  Not applicable ❒_5_ |
| --- | --- |

| 7. In the last 12 months, how often have you been denied health services, including dental care, because of your HIV status?  (Tick one box only.) | Never ❒_1_  Once ❒_2_  A few times ❒_3_  Often ❒_4_  Not applicable ❒_5_ |
| --- | --- |

| 8. In the last 12 months, have you been denied family planning services because of your HIV status?  (Tick one box only.) | Yes ❒_1_  No ❒_2_  Not applicable ❒^3^ |
| --- | --- |

| 9. In the last 12 months, have you been denied sexual and reproductive health services because of your HIV status? | Yes ❒_1_ | No ❒_2_ |
| --- | --- | --- |

***Section 2C: Internal stigma (the way you feel about yourself) and your fears***

| 1. In the last 12 months, have you experienced any of the following feelings because of your HIV status? (Tick one box for each category.) | | |
| --- | --- | --- |
| I feel ashamed | Yes ❒_1_ | No ❒_2_ |
| I feel guilty | Yes ❒_1_ | No ❒_2_ |
| I blame myself | Yes ❒_1_ | No ❒_2_ |
| I blame others | Yes ❒_1_ | No ❒_2_ |
| I have low self-esteem | Yes ❒_1_ | No ❒_2_ |
| I feel I should be punished | Yes ❒_1_ | No ❒_2_ |
| I feel suicidal | Yes ❒_1_ | No ❒_2_ |

| 2. In the last 12 months, have you done any of the following things because of your HIV status?  (Tick one box for each category). | | |
| --- | --- | --- |
| I have chosen not to attend social gathering(s) | Yes ❒_1_ | No ❒_2_ |
| I have isolated myself from my family and/or friends | Yes ❒_1_ | No ❒_2_ |
| I took the decision to stop working | Yes ❒_1_ | No ❒_2_ |
| I decided not to apply for a job/work or for a promotion | Yes ❒_1_ | No ❒_2_ |
| I withdrew from education/training or did not take up an opportunity  for education/ training | Yes ❒_1_ | No ❒_2_ |
| I decided not to get married | Yes ❒_1_ | No ❒_2_ |
| I decided not to have sex | Yes ❒_1_ | No ❒_2_ |
| I decided not to have (more) children | Yes ❒_1_ | No ❒_2_ |
| I avoided going to a local clinic when I needed to | Yes ❒_1_ | No ❒_2_ |
| I avoided going to a hospital when I needed to | Yes ❒_1_ | No ❒_2_ |

| 3. In the last 12 months, have you been *fearfu*l of any of the following things happening to you -whether or not they *actually* have happened to you? | | |
| --- | --- | --- |
| Being gossiped about | Yes ❒_1_ | No ❒_2_ |
| Being verbally insulted, harassed and/or threatened | Yes ❒_1_ | No ❒_2_ |
| Being physically harassed and/or threatened | Yes ❒_1_ | No ❒_2_ |
| Being physically assaulted | Yes ❒_1_ | No ❒_2_ |

| 4. In the last 12 months, have you been afraid that someone would not want to be sexually intimate with you because of your HIV-positive status? | Yes ❒_1_ | No ❒_2_ |
| --- | --- | --- |

***Section 2D: Rights, laws and policies***

| 1a. | | Have you heard of the *Declaration of Commitment on HIV/AIDS*, which protects the rights of people living with HIV? | Yes ❒_1_ | | | No ❒_2_ | |
| --- | --- | --- | --- | --- | --- | --- | --- |
| 1b. | If yes, have you ever read or discussed the content of this Declaration? | | Yes ❒_1_ | | | No ❒_2_ | |
|  |  | |  | | |  | |
| 2a. | Have you heard of *[insert the best-known national law/policy or set of guidelines from your country – choose one only],* which protect(s) the rights of people living with HIV in this country? | | Yes ❒_1_ | | | No ❒_2_ | |
| 2b. | If yes, have you ever read or discussed the content of this *[select the appropriate term based on your choice - law/policy/set of guidelines]*? | | Yes ❒_1_ | | | No ❒_2_ | |
|  |  | |  | | |  | |
| 3. | In the last 12 months, have any of the following things happened to you because of your HIV status? (Tick more than one box if appropriate). | | | | | |  |
|  | I was forced to submit to a medical or health procedure (including HIV testing) | | | | | | ❒_1_ |
|  | I was denied health insurance or life insurance because of my HIV status | | | | | | ❒_2_ |
|  | I was arrested or taken to court on a charge related to my HIV status | | | | | | ❒_3_ |
|  | I had to disclose my HIV status in order to enter another country | | | | | | ❒_4_ |
|  | I had to disclose my HIV status to apply for residence or nationality | | | | | | ❒_5_ |
|  | I was detained, quarantined, isolated or segregated | | | | | | ❒_6_ |
|  | None of these things happened to me | | | | | | ❒_7_ |
|  | |  |  | | |  | |
| 4a. | | In the last 12 months, have any of your rights as a person living with HIV been abused?  **If the answer is NO, please go to the next section (Section 2E: Effecting Change).** | Yes  ❒_1_ | No  ❒_2_ | Not sure  ❒^3^ | | |
| 4b. | If yes, have you tried to get legal redress for any abuse of your rights as a person living with HIV?  **If the answer is NO or NOT SURE, please go to question 4e.** | | Yes ❒_1_ | | | No ❒_2_ | |
| 4c. | Has this process begun in the last 12 months? | | Yes ❒_1_ | | | No ❒_2_ | |
| 4d. | What was the result? | | | | | |  |
| The matter has been dealt with | | | | | ❒_1_ | | |
| The matter is still in the process of being dealt with | | | | | ❒_2_ | | |
| Nothing happened/the matter was not dealt with | | | | | ❒_3_ | | |
| **Please now go to question 5a.** | | | | |  | | |

| 4e. If the response to Question 4b was **NO**, what was the reason for not trying to get legal redress? | |
| --- | --- |
|  | Insufficient financial resources to take action ❒_1_  Process of addressing the problem appeared too bureaucratic ❒_2_  Felt intimidated or scared to take action ❒_3_  Advised against taking action by someone else ❒_4_  No/little confidence that the outcome would be successful ❒_5_  None of the above ❒_6_ |

| 5a. | Have you tried to get a government employee(s) to take action against an abuse of your rights as a person living with HIV?  **If the answer is NO, please go to question 6a.** | Yes ❒_1_ | No ❒_2_ | |  |  |
| --- | --- | --- | --- | --- | --- | --- |
| 5b. | Did this happen in the last 12 months? | Yes ❒_1_ | No ❒_2_ | |  |  |
| 5c. | | What was the result? | | |  | |
|  | | The matter has been dealt with | | | ❒_1_ | |
|  | | The matter is still in the process of being dealt with | | | ❒_2_ | |
|  | | Nothing happened/the matter was not dealt with | | | ❒_3_ | |

| 6a. | Have you tried to get a local or national politician to take action against an abuse of your rights as a person living with HIV?  **If the answer is NO, please go to the next section (Section 2E: Effecting change).** | | Yes ❒_1_ | No ❒_2_ |
| --- | --- | --- | --- | --- |
| 6b. | | Did this happen in the last 12 months? | Yes ❒_1_ | No ❒_2_ |
| 6c. | | What was the result? | |  |
| The matter has been dealt with | | | | ❒_1_ |
| The matter is still in the process of being dealt with | | | | ❒_2_ |
| Nothing happened/the matter was not dealt with | | | | ❒_3_ |

***Section 2E: Effecting change***

| 1. In the last 12 months, have you confronted, challenged or educated someone who was stigmatizing and/or discriminating against you? | | Yes ❒_1_ | No ❒_2_ |  |  |
| --- | --- | --- | --- | --- | --- |
| 2a. Do you know of any organizations or groups that you can go to for help if you experience stigma or discrimination?  **If the answer is NO, please go to question 3.** | | Yes ❒_1_ | No ❒_2_ |  |  |
| 2b. If yes, which kinds of organizations or groups do you know about? (Tick more than one box if appropriate.) | | | | |  |
|  | People living with HIV support group ❒_1_  Network of people living with HIV ❒_2_  Local nongovernmental organization ❒_3_  Faith-based organization ❒_4_  A legal practice ❒_5_  A human rights organization ❒_6_  National nongovernmental organization ❒_7_  National AIDS council or committee ❒_8_  International nongovernmental organization ❒_9_  UN organization ❒_10_  Other ❒_11_ | | | |  |
| 2c. If you ticked **OTHER** please describe the kind of organization or group you are referring to: | | | | |  |
| 3. Have you sought help from any of the above organizations or groups to resolve an issue of stigma or discrimination? | | Yes ❒_1_ | No ❒_2_ | | |

4. If you have tried to resolve an issue of stigma and discrimination either on your own or with the assistance of others, briefly describe what the issue was about, who – if anyone - helped you, and how you and/or others tried to resolve the matter.

| **WHAT** was the issue of stigma and discrimination about? |
| --- |
| If others helped you resolve the matter – **WHO** helped you? |
| **HOW** did you (and, if appropriate, others) try to resolve the matter (i.e. what specifically did you and/or others do)? |

| 5a. In the last 12 months, have you supported other people living with HIV? | Yes ❒_1_ | No ❒_2_ |
| --- | --- | --- |
| 5b. If yes, what types of support did you provide (tick more than one box if appropriate)?  Emotional support (e.g. counselling, sharing personal stories and experiences) ❒_1_  Physical support (e.g. providing money or food, doing an errand for them) ❒_2_  Referral to other services ❒_3_ | | |

| 6. Are you currently a member of a people living with HIV support group and/or network? | | Yes ❒_1_ | No ❒_2_ | |
| --- | --- | --- | --- | --- |
| 7. In the last 12 months, have you been involved, either as a volunteer or as an employee, in any programme or project (either government or nongovernmental) that provides assistance to people living with HIV? | | Yes ❒_1_ | No ❒_2_ | |
| 8. In the last 12 months have you been involved in any efforts to develop legislation, policies or guidelines related to HIV? | | Yes ❒_1_ | No ❒_2_ | |
| 9. | Do you feel that you have the power to influence decisions in any of the following aspects…? (Tick *at least* one box. You can tick more than one if appropriate.) | | |  |
|  | Legal/rights matters affecting people living with HIV | | | ❒_1_ |
|  | Local government policies affecting people living with HIV | | | ❒_2_ |
|  | Local projects intended to benefit people living with HIV | | | ❒_3_ |
|  | National government policies affecting people living with HIV | | | ❒_4_ |
|  | National programmes/projects intended to benefit people living with HIV | | | ❒_5_ |
|  | International agreements/treaties | | | ❒_6_ |
|  | None of these things | | | ❒_7_ |
| 10. | There are a number of organizations of people living with HIV working against stigma and discrimination. If one of them asked you, **“What is the most important thing we should be doing as an organization to address stigma and discrimination?”** what would you recommend? (Tick only one box). | | |  |
|  | Advocating for the rights of all people living with HIV | | | ❒_1_ |
|  | Providing support to people living with HIV by providing emotional, physical and referral support | | | ❒_2_ |
|  | Advocating for the rights and/or providing support to particularly marginalized groups (men who have sex with men, injecting drug users, sex workers) | | | ❒_3_ |
|  | Educating people living with HIV about living with HIV (including treatment literacy) | | | ❒_4_ |
|  | Raising the awareness and knowledge of the public about AIDS | | | ❒_5_ |

***Section 3A: Testing/diagnosis***

| 1. Why were you tested for HIV? (Tick one or more boxes as appropriate). | |
| --- | --- |
| Employment | ❒_1_ |
| Pregnancy | ❒_2_ |
| To prepare for a marriage/sexual relationship | ❒_3_ |
| Referred by a clinic for sexually transmitted infections | ❒_4_ |
| Referred due to suspected HIV-related symptoms (e.g. tuberculosis) | ❒_5_ |
| Husband/wife/partner/family member tested positive | ❒_6_ |
| Illness or the death of husband/wife/partner/family member | ❒_7_ |
| I just wanted to know | ❒_8_ |
| Other  If you ticked **OTHER**, please describe what this reason was below: | ❒_9_ |

| 2. Was the decision to be tested for HIV up to you? (Tick only one box.) | |
| --- | --- |
| Yes, I took the decision myself to be tested (i.e. it was voluntary) | ❒_1_ |
| I took the decision to be tested, but it was under pressure from others | ❒_2_ |
| I was made to take an HIV test (coercion) | ❒_3_ |
| I was tested without my knowledge – I only found out after the test had been done | ❒_4_ |

| 3. Did you receive counselling when you were tested for HIV? (Tick only one box.) | |
| --- | --- |
| I received both pre- and post-HIV test counselling | ❒_1_ |
| I only received pre-test HIV counselling | ❒_2_ |
| I only received post-test HIV counselling | ❒_3_ |
| I did not receive any counselling when I had an HIV test | ❒_4_ |

***Section 3B: Disclosure and confidentiality***

| 1. For each of the following people or groups of people, please describe how they were first told about your HIV status, if they have been told.  Please tick your answers. Only tick more than one box in each line only if the answer is different for different individuals. | | | | | |
| --- | --- | --- | --- | --- | --- |
|  | **I told them** | **Someone else told them, WITH my consent** | **Someone else told them, WITHOUT my consent** | **They don’t know my HIV status** | **Not applicable** |
| Your husband/wife/partner | ❒_1_ | ❒_2_ | ❒_3_ | ❒_4_ | ❒_5_ |
| Other adult family members | ❒_1_ | ❒_2_ | ❒_3_ | ❒_4_ | ❒_5_ |
| Children in your family | ❒_1_ | ❒_2_ | ❒_3_ | ❒_4_ | ❒_5_ |
| Your friends/neighbours | ❒_1_ | ❒_2_ | ❒_3_ | ❒_4_ | ❒_5_ |
| Other people living with HIV | ❒_1_ | ❒_2_ | ❒_3_ | ❒_4_ | ❒_5_ |
| People who you work with (your co-workers) | ❒_1_ | ❒_2_ | ❒_3_ | ❒_4_ | ❒_5_ |
| Your employer(s)/boss(es) | ❒_1_ | ❒_2_ | ❒_3_ | ❒_4_ | ❒_5_ |
| Your clients | ❒_1_ | ❒_2_ | ❒_3_ | ❒_4_ | ❒_5_ |
| Injecting drug partners | ❒_1_ | ❒_2_ | ❒_3_ | ❒_4_ | ❒_5_ |
| Religious leaders | ❒_1_ | ❒_2_ | ❒_3_ | ❒_4_ | ❒_5_ |
| Community leaders | ❒_1_ | ❒_2_ | ❒_3_ | ❒_4_ | ❒_5_ |
| Health care workers | ❒_1_ | ❒_2_ | ❒_3_ | ❒_4_ | ❒_5_ |
| Social workers/counsellors | ❒_1_ | ❒_2_ | ❒_3_ | ❒_4_ | ❒_5_ |
| Teachers | ❒_1_ | ❒_2_ | ❒_3_ | ❒_4_ | ❒_5_ |
| Government officials | ❒_1_ | ❒_2_ | ❒_3_ | ❒_4_ | ❒_5_ |
| The media | ❒_1_ | ❒_2_ | ❒_3_ | ❒_4_ | ❒_5_ |

| 2a. How often did you feel pressure from other individuals living with HIV or from groups/networks of people living with HIV to disclose your HIV status? | Often  ❒_1_ | A few times  ❒_2_ | Once  ❒_3_ | Never  ❒_4_ |
| --- | --- | --- | --- | --- |
| 2b. How often did you feel pressure from other individuals **not** living with HIV (e.g. family members, social workers, nongovernmental organization employees) to disclose your HIV status? | Often  ❒_1_ | A few times  ❒_2_ | Once  ❒_3_ | Never  ❒_4_ |

| 3. Has a health care professional (for example, a doctor, nurse, counsellor, laboratory technician) ever told other people about your HIV status without your consent? | Yes ❒_1_ | No ❒_2_ | Not sure ❒_3_ |
| --- | --- | --- | --- |
|  |  |  |  |
| 4. How confidential do you think the medical records relating to your HIV status are? (Tick one box only). | | | |
| I am sure that my medical records will be kept completely confidential ❒_1_  I don’t know if my medical records are confidential ❒_2_  It is clear to me that my medical records are not being kept confidential ❒_3_ | | | |

| 5. How would you describe the reactions of these people (in *general*) when they first knew about your HIV status? Tick **one** number only for each category of people.  Tick **Not applicable** if these people do not know your HIV status or you don’t know what their reaction was. | | | | | | |
| --- | --- | --- | --- | --- | --- | --- |
|  | **Very discrimi-**  **natory** | **Discrimi-**  **natory** | **No different** | **Supportive** | **Very supportive** | **Not applicable** |
| Your husband/wife/ partner | ❒_1_ | ❒_2_ | ❒_3_ | ❒_4_ | ❒_5_ | ❒_6_ |
| Other adult family members | ❒_1_ | ❒_2_ | ❒_3_ | ❒_4_ | ❒_5_ | ❒_6_ |
| Children in your family | ❒_1_ | ❒_2_ | ❒_3_ | ❒_4_ | ❒_5_ | ❒_6_ |
| Your friends/neighbours | ❒_1_ | ❒_2_ | ❒_3_ | ❒_4_ | ❒_5_ | ❒_6_ |
| Other people living with HIV | ❒_1_ | ❒_2_ | ❒_3_ | ❒_4_ | ❒_5_ | ❒_6_ |
| Your co-workers | ❒_1_ | ❒_2_ | ❒_3_ | ❒_4_ | ❒_5_ | ❒_6_ |
| Your employer(s)/boss(es) | ❒_1_ | ❒_2_ | ❒_3_ | ❒_4_ | ❒_5_ | ❒_6_ |
| Your clients | ❒_1_ | ❒_2_ | ❒_3_ | ❒_4_ | ❒_5_ | ❒_6_ |
| Injecting drug partners | ❒_1_ | ❒_2_ | ❒_3_ | ❒_4_ | ❒_5_ | ❒_6_ |
| Religious leaders | ❒_1_ | ❒_2_ | ❒_3_ | ❒_4_ | ❒_5_ | ❒_6_ |
| Community leaders | ❒_1_ | ❒_2_ | ❒_3_ | ❒_4_ | ❒_5_ | ❒_6_ |
| Health care workers | ❒_1_ | ❒_2_ | ❒_3_ | ❒_4_ | ❒_5_ | ❒_6_ |
| Social workers/counsellors | ❒_1_ | ❒_2_ | ❒_3_ | ❒_4_ | ❒_5_ | ❒_6_ |
| Teachers | ❒_1_ | ❒_2_ | ❒_3_ | ❒_4_ | ❒_5_ | ❒_6_ |
| Government officials | ❒_1_ | ❒_2_ | ❒_3_ | ❒_4_ | ❒_5_ | ❒_6_ |
| The media | ❒_1_ | ❒_2_ | ❒_3_ | ❒_4_ | ❒_5_ | ❒_6_ |

| 6. Did you find the disclosure of your HIV status an empowering experience? (Tick **Not applicable** if you have not disclosed your HIV status). | |
| --- | --- |
| Yes | ❒_1_ |
| No | ❒_2_ |
| Not applicable | ❒_3_ |

***Section 3C: Treatment***

| 1. In general, how would you describe your health at the moment? (Tick only one box.) | | | | |
| --- | --- | --- | --- | --- |
| Excellent | | | | ❒_1_ |
| Very Good | | | | ❒_2_ |
| Good | | | | ❒_3_ |
| Fair | | | | ❒_4_ |
| Poor | | | | ❒_5_ |
|  | | | |  |
| 2a. Are you currently taking antiretroviral treatment?  (Tick only one box.) | Yes ❒_1_ | No ❒_2_ |  | |
| 2b. Do you have *access** to antiretroviral treatment, even if you are not currently taking it? (Tick only one box.) | Yes ❒_1_ | No ❒_2_ | Don’t know ❒_3_ | |
| 3a. Are you currently taking any medication to prevent or to treat opportunistic infections? (Tick only one box.) | Yes ❒_1_ | No ❒_2_ |  | |
| 3b. Do you have *access** to medication for opportunistic infections, even if you are not currently taking it? (Tick only one box.) | Yes ❒_1_ | No ❒_2_ | Don’t know ❒_3_ | |

* In this context **access** means that antiretroviral treatment is **available** and **free** or **you can afford it**.

| 4. In the last 12 months, have you had a constructive discussion with a health care professional(s) on the subject of your HIV-related treatment options? | Yes ❒_1_ | No ❒_2_ |
| --- | --- | --- |

| 5. In the last 12 months, have you had a constructive discussion with a health care professional(s) on other subjects such as your sexual and reproductive health, sexual relationship(s), emotional well-being, drug use, etc? | Yes ❒_1_ | No ❒_2_ |
| --- | --- | --- |

***Section 3D: Having children***

**Questions 1 – 4 can be completed by both male and female interviewees.**

| 1a. Do you have a child/children? | Yes ❒_1_ | No ❒_2_ |
| --- | --- | --- |
| 1b. If **Yes**, are any of these children known to be HIV-positive? | Yes ❒_1_ | No ❒_2_ |

| 2. Since being diagnosed as HIV-positive, have you ever received counselling about your reproductive options? | Yes ❒_1_ | No ❒_2_ | Not applicable ❒_3_ |
| --- | --- | --- | --- |

| 3. Has a health care professional ever advised you not to have a child since you were diagnosed as HIV-positive? | Yes ❒_1_ | No ❒_2_ | Not applicable ❒_3_ |
| --- | --- | --- | --- |

| 4. Has a health care professional ever coerced you into being sterilized since you were diagnosed as HIV-positive? | Yes ❒_1_ | No ❒_2_ | Not applicable ❒_3_ |
| --- | --- | --- | --- |

| 5. Is your ability to obtain antiretroviral treatment conditional on the use of certain forms of contraception? | Yes ❒_1_ | No ❒_2_ | Not applicable ❒_3_ | I don’t know ❒_4_ |
| --- | --- | --- | --- | --- |

**Questions 6 and 7 ought to be completed only by female interviewees.**

| 6. In the last 12 months, have you been coerced by a health care professional in relation to any of the following because of your HIV status? | | | |
| --- | --- | --- | --- |
| Termination of pregnancy (abortion) | Yes ❒_1_ | No ❒_2_ | Not applicable ❒_3_ |
| Method of giving birth | Yes ❒_1_ | No ❒_2_ | Not applicable ❒_3_ |
| Infant feeding practices | Yes ❒_1_ | No ❒_2_ | Not applicable ❒_3_ |

| 7a. Have you ever been given antiretroviral treatment to prevent mother-to-child transmission of HIV during pregnancy?  (Tick only one box.) | Yes - I have received such treatment ❒_1_  No - I did not know that such treatment existed ❒_2_  No - I was refused such treatment ❒_3_  No - I did not have access to such treatment ❒_4_  No - I was not HIV-positive when pregnant ❒_5_ | | |
| --- | --- | --- | --- |
| 7b. If yes, were you also given information about healthy pregnancy and motherhood as part of the programme to prevent mother-to-child transmission of HIV? | | Yes ❒_1_ | No ❒_2_ |

***Section 3E: Problems and challenges***

| **What do you see as the MAIN PROBLEMS and CHALLENGES in relation to:** |
| --- |
| **1. Testing and diagnosis:** |
| **2. Disclosure and confidentiality about being HIV-positive:** |
| **3. Antiretroviral treatment:** |
| **4. Having children when you are HIV-positive:** |

**Quality checks:** This section is designed to help the interviewer and team leader to check the questionnaire to make sure that it has been properly and fully completed. However, you also need to use your own judgement to make sure that a good job has been done! The team leader will check the interviewer’s responses on his/her return to base.

The interviewer should respond to the following points **before the closure of the interview** so that the interviewee is able to assist you to complete the responses to these questions.

| 1 | Has the interviewee answered **every** question in Sections 1-3 of the questionnaire? | Yes ❒ No ❒ |
| --- | --- | --- |
| If **No**, please specify which questions have not been answered and give reasons why: | | |
| 2 | Do the answers to Question 7 in Section 1 and Question 8 in Section 2A (groups the interviewee has belonged to or does belong to) appear consistent? If **No**, please explain: | Yes ❒ No ❒ |
| 3 | Does the information given in Section 1 (Questions 14 and 15) seem credible? (i.e. is the poverty level of the household roughly consistent with their experience of having insufficient money to buy food for the household – taking into account that some low-income households may grow their own food?)  If there are *differences*, have you checked the reasons with the interviewee and recorded why there are these differences below? | Yes ❒ No ❒  Yes ❒ |
| 4 | Has the front page of the questionnaire been completed? | Yes ❒ |
| **The last quality check can be completed by the interviewer after the interviewee has left, but** *before* **the interviewer leaves the place of the interview:** | | |
| 5 | Has the questionnaire code been written in the top right-hand corner of every page? | Yes ❒ |
