# Supplementary material for: Ebola virus disease-related stigma among survivors declined in Liberia over an 18-month, post-outbreak period: An observational cohort study
Source: PLoS Negl Trop Dis. 2019 Feb 27;13(2):e0007185. doi: 10.1371/journal.pntd.0007185 (PMC6411197; doi:10.1371/journal.pntd.0007185)
Supplement: S2 Appendix — (DOCX) [file pntd.0007185.s003.docx]

**S2 Appendix. Survey instrument for EVD-related stigma, sensitivity analyses, and multicollinearity assessment.**

**Table A: Stigma questionnaire administered to participants at initial visit and visit 4.**

| **Read the following questions to the volunteer and ask which apply to their experience** | No | Yes |
| --- | --- | --- |
| 1. Forced to change residence because of social alienation from family and/or friends. |  |  |
| 2. Lost a job or another source of income because of being infected. |  |  |
| 3. Lost a spouse because of fear of being infected from personal interaction. |  |  |
| 4. Deprived from attending gathering (e.g., school, church, social) for fear of infecting others. |  |  |
| 5. Isolated yourself from family and/or friends. |  |  |
| 6. Withdrew from education/training or did not take up an opportunity for education/training |  |  |
| 7. Afraid that someone would not want to be sexually intimate with you as a survivor. |  |  |

**Table B: Associations between predictors and stigma at initial visit (linear model)**

|  | Estimated Stigma Increase  (95% CI) | p−value |
| --- | --- | --- |
| Education (Primary, junior high or vocational)^1^ | 0.42 (0.14, 0.7) | <0.005 |
| Education (High school or beyond)^1^ | 0.18 (-0.11, 0.46) | 0.22 |
| Age (12-19)^2^ | -0.81 (-1.12, -0.49) | <0.005 |
| Age (30-39)^2^ | 0.1 (-0.16, 0.36) | 0.45 |
| Age (40-49)^2^ | -0.14 (-0.44, 0.16) | 0.36 |
| Age (50+)^2^ | -0.48 (-0.83, -0.13) | 0.01 |
| Gender (Female) | 0.06 (-0.15, 0.26) | 0.6 |
| Hospitalized Since Acute EVD | 0.11 (-0.43, 0.65) | 0.69 |
| Referred to Medical Care | 0.33 (0.14, 0.53) | <0.005 |
| Pregnant | -0.22 (-0.63, 0.19) | 0.3 |
| Infection Source (Family)^3^ | 0.03 (-0.23, 0.3) | 0.8 |
| Infection Source (Job related)^3^ | 0.24 (-0.18, 0.66) | 0.27 |

1. No formal education is comparison group for education.
2. Age 20-29 is comparison group for age.
3. Other/unknown is the comparison group for infection source.

**Table C: Associations between predictors and stigma at initial visit (poisson model)**

|  | Estimated Relative Risk  (95% CI) | p−value |
| --- | --- | --- |
| Education (Primary, junior high or vocational)^1^ | 1.38 (1.15, 2.02) | <0.005 |
| Education (High school or beyond)^1^ | 1.16 (0.9, 1.58) | 0.22 |
| Age (12-19)^2^ | 0.49 (0.33, 0.61) | <0.005 |
| Age (30-39)^2^ | 1.06 (0.85, 1.43) | 0.45 |
| Age (40-49)^2^ | 0.91 (0.64, 1.18) | 0.36 |
| Age (50+)^2^ | 0.68 (0.43, 0.88) | 0.01 |
| Gender (Female) | 1.04 (0.86, 1.3) | 0.6 |
| Hospitalized Since Acute EVD | 1.09 (0.65, 1.91) | 0.69 |
| Referred to Medical Care | 1.27 (1.15, 1.7) | <0.005 |
| Pregnant | 0.85 (0.53, 1.21) | 0.3 |
| Infection Source (Family)^3^ | 1.03 (0.79, 1.35) | 0.8 |
| Infection Source (Job related)^3^ | 1.17 (0.83, 1.94) | 0.27 |

1. No formal education is comparison group for education.
2. Age 20-29 is comparison group for age.
3. Other/unknown is the comparison group for infection source.

**Table D: Results from logistic regression for each individual stigma item and age group controlling for all other covariates**

|  | Age Range1 | Odds Ratio (95% CI) | p-value |
| --- | --- | --- | --- |
| Stigma Item 1 | 12-19 | 0.77 (0.46, 1.28) | 0.32 |
|  | 30-39 | 0.94 (0.63, 1.41) | 0.76 |
|  | 40-49 | 0.35 (0.19, 0.61) | <0.005 |
|  | 50+ | 0.4 (0.21, 0.76) | 0.01 |
| Stigma Item 2 | 12-19 | 0.08 (0.03, 0.18) | <0.005 |
|  | 30-39 | 1.68 (1.16, 2.46) | 0.01 |
|  | 40-49 | 1.29 (0.83, 2.02) | 0.25 |
|  | 50+ | 0.89 (0.52, 1.5) | 0.66 |
| Stigma Item 3 | 12-19 | 0.08 (0.01, 0.29) | <0.005 |
|  | 30-39 | 0.84 (0.46, 1.53) | 0.57 |
|  | 40-49 | 0.8 (0.39, 1.57) | 0.52 |
|  | 50+ | 0.12 (0.02, 0.41) | <0.005 |
| Stigma Item 4 | 12-19 | 0.58 (0.26, 1.23) | 0.17 |
|  | 30-39 | 1.19 (0.68, 2.07) | 0.55 |
|  | 40-49 | 0.91 (0.45, 1.77) | 0.78 |
|  | 50+ | 0.8 (0.33, 1.78) | 0.6 |
| Stigma Item 5 | 12-19 | 0.72 (0.41, 1.27) | 0.26 |
|  | 30-39 | 0.98 (0.61, 1.55) | 0.92 |
|  | 40-49 | 1.05 (0.61, 1.77) | 0.87 |
|  | 50+ | 1.04 (0.55, 1.93) | 0.89 |
| Stigma Item 6 | 12-19 | 0.66 (0.33, 1.29) | 0.24 |
|  | 30-39 | 0.61 (0.35, 1.04) | 0.07 |
|  | 40-49 | 0.68 (0.36, 1.27) | 0.24 |
|  | 50+ | 0.27 (0.08, 0.74) | 0.02 |
| Stigma Item 7 | 12-19 | 0.29 (0.14, 0.57) | <0.005 |
|  | 30-39 | 1.26 (0.83, 1.94) | 0.28 |
|  | 40-49 | 1.21 (0.73, 1.99) | 0.46 |
|  | 50+ | 0.63 (0.31, 1.2) | 0.17 |

1. Age 20-29 is comparison group for age.

**Table E: Results from logistic regression for each individual stigma item and education controlling for all other covariates**

|  | Education Level1 | Odds Ratio (95% CI) | p-value |
| --- | --- | --- | --- |
| Stigma Item 1 | Primary, junior high or vocational | 1.31 (0.81, 2.14) | 0.28 |
|  | High school or beyond | 1.2 (0.74, 1.97) | 0.46 |
| Stigma Item 2 | Primary, junior high or vocational | 1.2 (0.78, 1.84) | 0.41 |
|  | High school or beyond | 0.91 (0.59, 1.4) | 0.66 |
| Stigma Item 3 | Primary, junior high or vocational | 0.99 (0.51, 1.95) | 0.98 |
|  | High school or beyond | 0.51 (0.25, 1.05) | 0.06 |
| Stigma Item 4 | Primary, junior high or vocational | 1.82 (0.95, 3.64) | 0.08 |
|  | High school or beyond | 1.47 (0.76, 2.99) | 0.26 |
| Stigma Item 5 | Primary, junior high or vocational | 2.03 (1.22, 3.44) | 0.01 |
|  | High school or beyond | 1.2 (0.71, 2.07) | 0.51 |
| Stigma Item 6 | Primary, junior high or vocational | 4.88 (1.86, 16.82) | <0.005 |
|  | High school or beyond | 6.76 (2.62, 23.06) | <0.005 |
| Stigma Item 7 | Primary, junior high or vocational | 1.72 (1.04, 2.89) | 0.04 |
|  | High school or beyond | 1.28 (0.77, 2.17) | 0.34 |

1. No formal education is comparison group for education.

**Table F:  Associations between predictors and stigma at initial visit (excluding stigma item 6)**

|  | Estimated Odds Ratio  (95% CI) | p-value |
| --- | --- | --- |
| Education (Primary, junior high or vocational)^1^ | 1.76 (1.23, 2.55) | <0.005 |
| Education (High school or beyond)^1^ | 1.09 (0.76, 1.57) | 0.65 |
| Age (12-19)^2^ | 0.29 (0.19, 0.45) | <0.005 |
| Age (30-39)^2^ | 1.28 (0.92, 1.78) | 0.15 |
| Age (40-49)^2^ | 0.95 (0.65, 1.41) | 0.81 |
| Age (50+)^2^ | 0.62 (0.39, 0.97) | 0.04 |
| Gender (Female) | 1.19 (0.9, 1.55) | 0.22 |
| Hospitalized Since Acute EVD | 1.13 (0.56, 2.27) | 0.72 |
| Referred to Medical Care | 1.5 (1.16, 1.94) | <0.005 |
| Pregnant | 0.83 (0.49, 1.4) | 0.5 |
| Infection Source (Family)^3^ | 1.11 (0.79, 1.57) | 0.55 |
| Infection Source (Job related)^3^ | 1.55 (0.9, 2.66) | 0.11 |

1. No formal education is comparison group for education.
2. Age 20-29 is comparison group for age.
3. Other/unknown is the comparison group for infection source.

**Table G: Initial assessment associations between predictors and stigma excluding HIV positive participants**

|  | Estimated Odds Ratio  (95% CI) | p-value |
| --- | --- | --- |
| Education (Primary, junior high or vocational)^1^ | 1.85 (1.28, 2.67) | <0.005 |
| Education (High school or beyond)^1^ | 1.15 (0.8, 1.66) | 0.45 |
| Age (12-19)^2^ | 0.32 (0.21, 0.49) | <0.005 |
| Age (30-39)^2^ | 1.2 (0.86, 1.67) | 0.28 |
| Age (40-49)^2^ | 0.88 (0.59, 1.3) | 0.51 |
| Age (50+)^2^ | 0.56 (0.35, 0.87) | 0.01 |
| Gender (Female) | 1.16 (0.89, 1.53) | 0.27 |
| Hospitalized Since Acute EVD | 0.98 (0.48, 1.98) | 0.96 |
| Referred to Medical Care | 1.5 (1.16, 1.95) | <0.005 |
| Pregnant | 0.73 (0.43, 1.22) | 0.23 |
| Infection Source (Family)^3^ | 1.04 (0.74, 1.47) | 0.83 |
| Infection Source (Job related)^3^ | 1.28 (0.74, 2.21) | 0.38 |

1. No formal education is comparison group for education.
2. Age 20-29 is comparison group for age.
3. Other/unknown is the comparison group for infection source.

**Table H: Generalized Variance Inﬂation Factors (GVIF) for covariates used in generalized mixed effects model (Table 3)**

|  | GVIF |
| --- | --- |
| 18-Month Visit | 1.02 |
| Pregnant | 1.07 |
| Woman of Reproductive Age | 1.09 |
| HIV Positive | 1 |

**Table I: Generalized Variance Inﬂation Factors (GVIF) for covariates used in ordinal logistic regression model (Table 4)**

|  | GVIF | Degrees of Freedom | GVIF1/(2∗DF ) |
| --- | --- | --- | --- |
| Education Level | 1.46 | 2 | 1.1 |
| Age Range | 1.45 | 4 | 1.05 |
| Gender | 1.2 | 1 | 1.1 |
| Hospitalized Since Acute EVD | 1.03 | 1 | 1.01 |
| Referred to Medical Care | 1.06 | 1 | 1.03 |
| Pregnancy | 1.09 | 1 | 1.04 |
| EVD Source | 1.15 | 2 | 1.04 |
